# Genomic diversity and molecular epidemiology of *Pasteurella multocida*

**DOI:** 10.1371/journal.pone.0249138

**Published:** 2021-04-06

**Authors:** Emily Smith, Elizabeth Miller, Jeannette Munoz Aguayo, Cristian Flores Figueroa, Jill Nezworski, Marissa Studniski, Ben Wileman, Timothy Johnson

**Affiliations:** 1 Department of Veterinary and Biomedical Sciences, University of Minnesota, Saint Paul, MN, United States of America; 2 Mid-Central Research and Outreach Center, University of Minnesota, Willmar, Minnesota, United States of America; 3 Blue House Veterinary LLC, Buffalo Lake, Minnesota, United States of America; 4 Select Genetics, Willmar, MN, United States of America; Cornell University, UNITED STATES

## Abstract

*Pasteurella multocida* is a bacterial pathogen with the ability to infect a multitude of hosts including humans, companion animals, livestock, and wildlife. This study used bioinformatic approaches to explore the genomic diversity of 656 *P*. *multocida* isolates and epidemiological associations between host factors and specific genotypes. Isolates included in this study originated from a variety of hosts, including poultry, cattle, swine, rabbits, rodents, and humans, from five different continents. Multi-locus sequence typing identified 69 different sequence types. *In-silico* methodology for determining capsular serogroup was developed, validated, and applied to all genome sequences, whereby capsular serogroups A, B, D, and F were found. Whole genome phylogeny was constructed from 237,670 core single nucleotide variants (SNVs) and demonstrated an overall lack of host or capsular serogroup specificity, with the exception of isolates from bovine sources. Specific SNVs within the *srlB* gene were identified in *P*. *multocida* subsp. *septica* genomes, representing specific mutations that may be useful for differentiating one of the three known subspecies. Significant associations were identified between capsular serogroup and virulence factors, including capsular serogroup A and OmpH1, OmpH3, PlpE, and PfhB1; capsular serogroup B and HgbA and PtfA; and capsular serogroup F and PtfA and PlpP. Various mobile genetic elements were identified including those similar to ICE*Pmu*1, ICE*hin1056*, and IncQ1 plasmids, all of which harbored multiple antimicrobial resistance-encoding genes. Additional analyses were performed on a subset of 99 isolates obtained from turkeys during fowl cholera outbreaks from a single company which revealed that multiple strains of *P*. *multocida* were circulating during the outbreak, instead of a single, highly virulent clone. This study further demonstrates the extensive genomic diversity of *P*. *multocida*, provides epidemiological context to the various genotyping schemes that have traditionally been used for differentiating isolates, and introduces additional tools for *P*. *multocida* molecular typing.

## Introduction

*Pasteurella multocida* is a zoonotic pathogen with transboundary dissemination across a multitude of hosts and geographic landscapes. Infections with this Gram-negative bacterium are responsible for significant morbidity and mortality in both humans and animals, as well as economic losses in the livestock industry. Common disease manifestations due to *P*. *multocida* include fowl cholera in poultry, hemorrhagic septicemia in cattle, and atrophic rhinitis in swine [1,2]. Human infections are typically the result of bites from cats or dogs, most of which result in mild wound infections, but complications such as osteomyelitis or septicemia can occur [3–6].

Previous studies have demonstrated that poor biosecurity or management practices have contributed to *P*. *multocida* outbreaks in poultry, cattle, and swine [7–9]. Other risk factors, such as co-infection with another pathogenic organism, age, and vaccination status of the animals have also been reported [10–14]. In commercial turkey production, it is mostly unknown whether outbreaks of fowl cholera are attributable to a highly virulent single clone, or whether multiple *P*. *multocida* strains are circulating simultaneously. The identification of multiple strains circulating at the same time may further emphasize the role of biosecurity in prevention of fowl cholera, however, there has been little effort to determine specific strains responsible for outbreaks at the whole-genome level.

To date, typing schemes that target specific genotypes or phenotypes are primarily utilized to classify *P*. *multocida* strains. Specifically, *P*. *multocida* can be classified into 5 capsular serogroups (A, B, D, E, and F) based on the antigenicity of the outer capsule [15–17]. This capsule plays a major role in the overall virulence of *P*. *multocida*, as isolates lacking this capsule demonstrate reduced capacity to invade and adhere to host cells and resist phagocytosis [18–20]. *P*. *multocida* can be further divided into 16 somatic serovars using the Heddleston serotyping scheme based on the structure of lipopolysaccharide antigens [21,22]. Although this serotyping scheme is widely used, many isolates are non-typable with this method and results are often ambiguous due to cross-reactivity during the gel diffusion precipitin test [23–26]. More recently, a multiplex PCR assay has been developed that is able to distinguish Heddleston serotypes of *P*. *multocida* based on the genetic organization of the LPS outer core biosynthesis loci with greater accuracy than the traditional gel diffusion precipitin test. [23,27]. Overall, serotyping can provide useful information about the cell-surface antigens of *P*. *multocida*. However, it is not an indicator of genetic diversity between isolates and has limited capacity to differentiate related strains.

*P*. *multocida* isolates can also be classified into one of three subspecies, subsp. *multocida*, *septica*, or *gallicida* based on the ability to ferment sorbitol and dulcitol [28]. Some previous studies have stated that *P*. *multocida* subsp. *multocida* and *septica* are associated with more severe clinical presentations, but these studies have been limited in scope and sample size [29,30]. Phenotypic methods have traditionally been used to determine subspecies, but these methods are time consuming and resource intensive. A PCR assay has been developed to distinguish subspecies based on the 16S rRNA gene, however, specific genes associated with sorbitol or dulcitol fermentation that may differentiate subsp. *multocida*, *septica*, and *gallicida* have not yet been identified [31]. Determining these genetic components that differentiate subspecies would allow for more rapid identification and lay a foundation for studies on epidemiological factors that may be associated with specific subspecies.

Multi-locus sequence typing (MLST) is another common method used to differentiate *P*. *multocida* strains and is based on the allelic profiles of seven housekeeping genes [32]. There are two different schemes available for MLST of *P*. *multocida*: the Rural Industries Research and Development Organization (RIRDC) scheme, which currently includes 373 different sequence types, and the Multi-host scheme, which currently consists of 127 different sequence types [33–35]. Both schemes have been widely used to sequence type isolates from various hosts, collection dates, and geographic regions [36–41]. The use of MLST alone excludes the possibility of discovering variation that exists outside seven specific genes. Therefore, it is often used in combination with other genotypic methods, such as the identification of virulence factors or antimicrobial resistance genes, to provide a more complete picture of the *P*. *multocida* study population [36,42–45]. Multiple plasmids containing antimicrobial resistance genes have also been identified in *P*. *multocida* isolates from multiple hosts and geographic regions, some of which are highly similar to those identified in other microbial species [46–48].

While the various typing schemes and assays for differentiating *P*. *multocida* isolates have enhanced the understanding of underlying molecular mechanisms and genetic diversity of *P*. *multocida*, they alone have little value in the absence of epidemiological data. Additionally, the associations between these different typing methods and relationships with host and geography have not been fully explored. Therefore, the purpose of this study was to characterize all publicly available *P*. *multocida* genomes and their associated raw reads using bioinformatic and epidemiologic approaches to provide insight into the diversity of the pathogen population with respect to host, geography, and genetic indicators of virulence and antimicrobial resistance. This study also employed the application of genomic tools in outbreak investigations, using fowl cholera outbreaks as an example to highlight the genetic diversity of *P*. *multocida* among farms from a single commercial turkey producer.

## Results

There were a total of 656 *P*. *multocida* whole genome sequences included in this study. These sequences were extracted from the NCBI Short Read Archive database (*n* = 338), NCBI Assembly database (*n* = 193), and from archived isolates at the University of Minnesota Mid-Central Research and Outreach Center (*n* = 125). The genomes were sourced from isolates from a variety of hosts, a majority of which were avian in origin, followed by bovine, rabbit, and swine species (Table 1). Most genomes came from isolates from hosts in North America (*n* = 390), but isolates from Asia (*n* = 98), Europe (*n* = 42), Australia (*n* = 95), and South America (*n* = 6) were also present. Collection dates ranged from 1936 to 2019. The Genbank assembly accession numbers and SRA numbers of genomes used in this study and accompanying metadata can be found in S1 Dataset.

**Table 1:** Hosts from which *P*. *multocida* was isolated (*n* = 656).

Host	No.	%	Specific host
Avian	373	56.3	Chicken (130); Duck (10); Goose (3); Gull (1); Pine siskin (1); Turkey (225)*; Unknown (3)
Bovine	155	23.5	Bison (3); Buffalo (30); Cattle (123)
Camelid	2	0.3	Alpaca (2)
Canine	2	0.3	Dog (1); Unknown (1)
Caprine	2	0.3	Goat (2)
Cervid	3	0.5	Deer (3)
Feline	2	0.3	Cat (2)
Human	16	2.4	Human (16)
Lagomorph	35	5.3	Rabbit (35)
Marsupial	14	2.1	Rufous rat-kangaroo (1); Squirrel glider (11); Woylie (2)
Otariid	6	0.9	Australasian fur seal (4); Subantarctic fur seal (2)
Ovine	4	0.6	Sheep (4)
Rodent	2	0.3	Unknown (2)
Swine	21	3.2	Boar (5); Pig (16)
Unknown	19	2.9	Unknown (19)

*125 isolates were provided by the University of Minnesota Mid-Central Research and Outreach Center for the current study.

### Sequence-typing demonstrates broad host range and presence of novel sequence types

Amongst the *P*. *multocida* genomes, 69 different sequence types (STs) were identified. Some STs appeared to be host-specific, such as the 75 genomes identified as ST20 that were exclusively from chickens in Australia. Similarly, ST79 included 99 genomes of bovine origin from North America, Asia, and Europe. However, many STs were not comprised of genomes from a single host species. For example, 36 genomes were identified as ST9, which has a broad host range amongst the genomes in this study, including avian, bovine, feline, rabbit, rodent, and swine sources. Of the 16 *P*. *multocida* genomes from humans, 13 fell into STs that included other hosts, such as ST6, which also included genomes from poultry or canine sources, and ST25, which included 4 genomes from human wounds and abscesses, but also included two genomes from turkeys. Additionally, ST242 contained 3 *P*. *multocida* genomes from human septicemia patients, but also included genomes from chickens and turkeys. There were 114 genomes that were unable to be sequence-typed. Of these, 110 (96.5%) genomes had all seven alleles identified, so it is likely that these represent novel sequence types that did not exist under the current scheme. The collection dates for genomes unable to be sequence-typed ranged from 1970 to 2018.

### *In-silico* capsular serotyping identifies minimal differences in host range and geographic distribution

An *in-silico* capsular serogrouping method was developed to identify the presence of genes encoding *P*. *multocida* capsular antigens A, B, D, and F within *P*. *multocida* genomes. There were 55 *P*. *multocida* genomes that had capsular serogroup data available in NCBI. The *in-silico* capsular serogrouping method correctly determined the capsular serogroup for 54 (98.1%) of these genomes, demonstrating that the *in-silico* methodology developed in this study may be a useful tool for capsular serogroup prediction when other methods are unavailable. The one genome for which the *in-silico* method did not correctly identify the capsular serogroup was designated as capsular serogroup A in the NCBI Assembly database but was identified as serogroup F using the *in-silico* methodology. When applied to all 656 genomes, capsular serogroups A (*n* = 516), B (*n* = 45), D (*n* = 33), and F (*n* = 62) were identified. Among the genomes in this study, 482 (73.5%) had >50% coverage of the *cap* gene used to distinguish capsular serogroups. The proportion of *P*. *multocida* genomes designated as different capsular serogroups within each host category were identified (Table 2). Capsular serogroup E was not identified from any genomes in this study. Capsular serogroup A was found in every host category with the exception of caprine hosts. All four capsular serogroups were found in isolates of avian origin, but capsular serogroup A (85.0%) was most common. For *P*. *multocida* isolates of bovine origin, capsular serogroup A (71.0%) was the most common, followed by capsular serogroups B (27.1%) and F (1.9%). Similarly, for isolates of swine origin, capsular serogroup A (66.7%) was the most common, followed by capsular serogroups D (19.0%), F (9.5%), and B (4.8%). All *P*. *multocida* isolates from human isolates were from serogroup A, with the exception of one which was of capsular serogroup F. The A and F capsular serogroups were also the only serogroups identified in cats and dogs. There did not appear to be major differences in the geographic distribution of *P*. *multocida* capsular serogroups. Capsular serogroups A and F were identified in isolates from all continents. Capsular serogroups B and D were only found in isolates from Asia, Europe, and North America, but this is likely reflective of the small number of isolates that were identified as these two capsular serogroups.

**Table 2:** *Pasteurella multocida* capsular serotypes by host category (*n* = 656).

Species	A (*n* = 516)	B (*n* = 45)	D (*n* = 33)	F (*n* = 62)
Avian	317 (85.0)	2 (0.5)	24 (6.4)	30 (8.0)
Bovine	110 (71.0)	42 (27.1)	0 (0.0)	3 (1.9)
Camelid	2 (100.0)	0 (0.0)	0 (0.0)	0 (0.0)
Canine	1 (50.0)	0 (0.0)	0 (0.0)	1 (50.0)
Caprine	0 (0.0)	0 (0.0)	2 (100.0)	0 (0.0)
Cervid	3 (100.0)	0 (0.0)	0 (0.0)	0 (0.0)
Feline	1 (50.0)	0 (0.0)	0 (0.0)	1 (50.0)
Human	15 (93.8)	0 (0.0)	0 (0.0)	1 (6.3)
Lagomorph	19 (54.3)	0 (0.0)	2 (5.7)	14 (40.0)
Marsupial	13 (92.9)	0 (0.0)	0 (0.0)	1 (7.1)
Otariid	6 (100.0)	0 (0.0)	0 (0.0)	0 (0.0)
Ovine	2 (50.0)	0 (0.0)	0 (0.0)	2 (50.0)
Rodent	1 (50.0)	0 (0.0)	0 (0.0)	1 (50.0)
Swine	14 (66.7)	1 (4.8)	4 (19.0)	2 (9.5)
Unknown	12 (62.3)	0 (0.0)	1 (5.3)	6 (31.6)

The numbers in parentheses reflect the percentage of genomes from the corresponding host type that belong to that particular serogroup.

### SNV-based phylogeny demonstrates apparent bovine host specificity, but lack of host specificity for other sources

**Fig 1:**
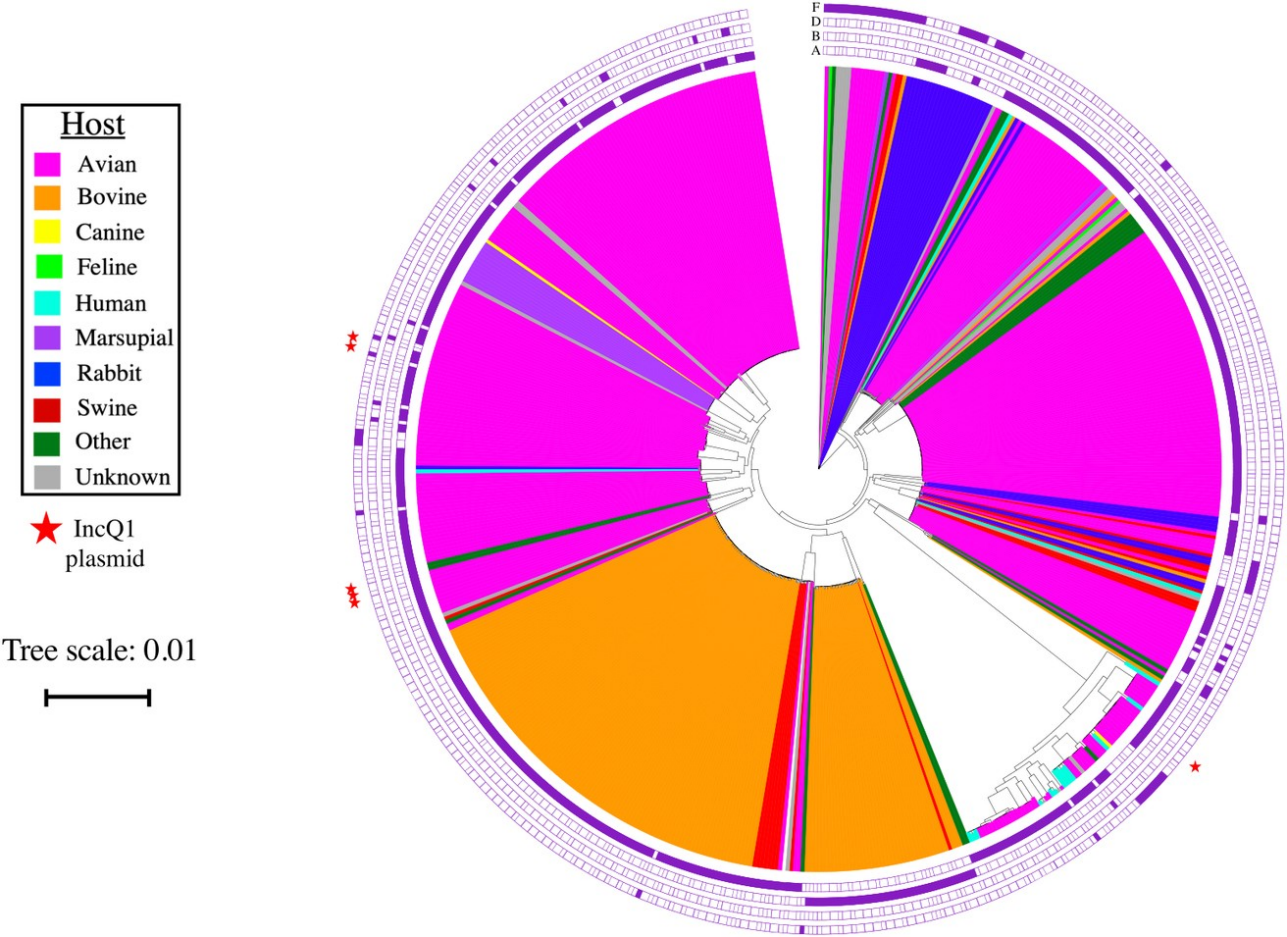
SNV-based phylogeny of *Pasteurella multocida*. Phylogenetic tree constructed from 237,670 core SNVs identified from 653 whole-genome sequences. The colors inside the tree represent different host sources (see host legend), the outer rings represent the corresponding capsular serogroup (filled = present and white = absent), and the star represents the presence of the IncQ1 plasmid (star = present and none = absent). The scale bar represents a branch length of 0.01 (approximately 2,377 SNVs).

There were 237,670 core single nucleotide variants (SNVs) identified from 653 genome sequences. One genome (SRR2907037) was removed from the SNV alignment because no SNVs were identified despite 98.1% of nucleotides aligning to the reference genome (GCA_000754275.1), and three genome sequences were automatically removed from the SNV alignment by Gubbins due to a high proportion of missing data (SRR5256406, PP64, PP74). The transversion model of DNA substitution with empirical base frequencies and three rate categories was selected as the best fit model by IQ-TREE and was used to construct the phylogenetic tree (Fig 1).

Almost all of the isolates from bovine sources fell into two phylogenetic clades (Fig 1), potentially indicating some degree of host-specificity. This includes all of the ST79 (*n* = 99) and ST122 (*n* = 42) isolates of primarily bovine origin. The majority of ST79 genomes belonged to capsular serogroup A, while all of the ST122 genomes belonged to capsular serogroup B. Four of the ST122 isolates were bovine vaccine strains of *P*. *multocida*, while the rest were recovered from sources including the nasal cavity, throat, lungs, blood, and bone marrow of buffalo and cattle from different continents, with the exception of one isolate of swine origin. In contrast, the isolates of avian origin were dispersed throughout the tree. This may potentially indicate that birds are susceptible to a wider variety of *P*. *multocida* strains compared to bovine hosts. While there is some evidence of clustering of the isolates from rabbits and swine, these clusters were also dispersed throughout the tree. There did not appear to be any major patterns on the phylogenetic tree by collection year.

Interestingly, there was one clade with a much longer branch length compared to the rest of the clades in the phylogenetic tree that represents more than 6,000 SNV differences from all other clades. This outer clade contains 13 of the 16 *P*. *multocida* isolates from humans, as well as isolates of avian, canine, and rodent origin. Capsular serogroups A, D, and F were found in the isolates from this clade. Isolates from this clade originated from Asia, Europe, and North America. Subspecies information was only available for five of these isolates, two of which were subsp. *multocida*, and three of which were subsp. *septica*. Upon further investigation of what may differentiate this clade from other genomes, 1,911 genes and gene groups were identified with significantly increased or decreased odds of being present in this specific clade compared to the rest of the *P*. *multocida* genomes. Genomes in this clade had significantly increased odds of containing *esiB* (OR = 6.1; *p* = 0.022), annotated as a secretory immunoglobulin A-binding gene, which has been shown to impair host neutrophil activation, and toxin *cdiA* (OR = 15.8; *p* = 0.009), which has been shown to demonstrate contact-dependent growth inhibition of surrounding bacteria [49,50]. The *oatA* gene encoding an O-acetyltransferase, which allows the bacteria to evade an initial host immune response, was also significantly associated with outer clade (OR = infinite; *p* = 0.003) [51]. Genes associated with antimicrobial resistance were also found at higher prevalence in this clade compared to all other genomes, including *etk* (OR = 14.7; *p* = 0.002), a putative tryosine-protein kinase that has been associated with resistance to polymyxin, as well as *tetD* (OR = 100.5; *p*<0.0001), a tetracycline efflux gene [52]. Additionally, the outer clade had significantly decreased odds of containing *wzc* (OR = 0.09; *p* = 0.02), a putative tyrosine-protein kinase associated with susceptibility to macrolide antibiotics [53].

### SNV-based phylogeny shows persistence of multiple strains involved in fowl cholera outbreaks

**Fig 2:**
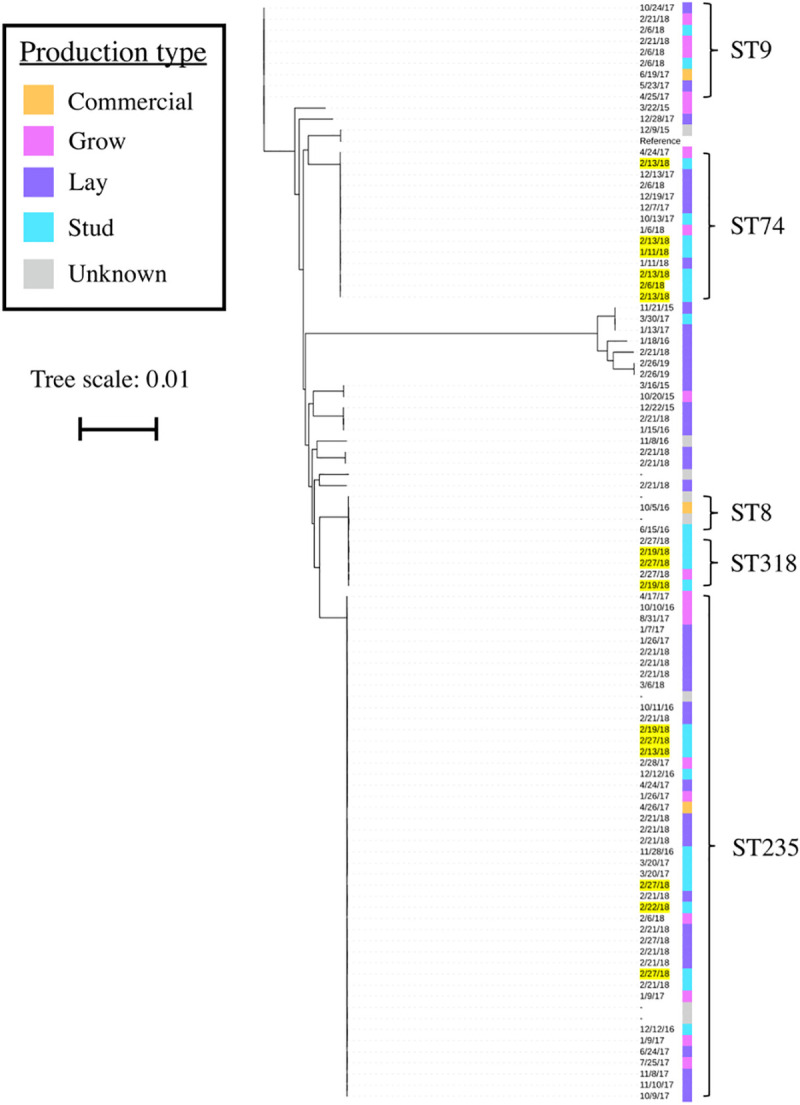
SNV-based phylogeny of *P*. *multocida* isolates involved in recurrent fowl cholera outbreaks within the same commercial turkey company. Phylogenetic tree constructed from 100,023 core SNVs of 99 *P*. *multocida* genomes. These genomes originated from clinical isolates collected during multiple fowl cholera outbreaks within a single company. The different colors represent different stages of turkey production (see legend). Collection dates are listed, and those highlighted in yellow represent successive samplings from a single indoor flock during an outbreak of fowl cholera in 2018. Clusters of specific sequence types are indicated with brackets. The scale bar represents a branch length of 0.01 (approximately 1,000 SNVs).

A collection of 99 *P*. *multocida* isolates involved in outbreaks of fowl cholera within a single vertically integrated commercial turkey production company occurring over five years was investigated. This subset includes genomes from all four capsular serogroups identified *in-silico*. A total of 100,023 SNVs were identified amongst the genomes of these isolates, indicating that multiple clones were implicated in this persistent and recurring disease issue. The core SNV phylogeny did not demonstrate associations between different stages of production, indicating that birds of all ages were likely susceptible to the same strains of *P*. *multocida* (Fig 2). Although collection dates ranged from 2015 to 2019, there were no distinct patterns in collection date on the phylogenetic tree, demonstrating that similar strains of *P*. *multocida* have persisted on farms from the same company over time, or were re-introduced from an external source on multiple occasions. Additionally, isolates of 3 different sequence types were collected from a single flock that experienced a fowl cholera outbreak in early 2018, indicating that multiple strains of *P*. *multocida* may be involved in the same fowl cholera outbreak within a flock.

### Pangenome analyses reveals key genes associated with *P*. *multocida* subspecies

There were 68 genomes for which subspecies information was available on NCBI, which included subsp. *multocida* (*n* = 48), subsp. *septica* (*n* = 16), and subsp. *gallicida* (*n* = 4). The genomes in each of these subspecies were comprised of multiple host sources, capsular serogroups, and sequence types. A search of the *P*. *multocida* pangenome, consisting of 37,854 genes, revealed the presence of sorbitol-specific PTS enzymes encoded by the *srl* operon, *srlAEBD*, which is homologous to the *gut* operon in *E*. *coli*, in 95.4% of *P*. *multocida* genomes [54]. This included 13 genomes of the *septica* subspecies, which are distinguished based on an inability to ferment sorbitol [54,55]. As no other genes associated with sorbitol fermentation were identified, this operon was further investigated in a SNV analysis of the 68 genomes of known subspecies. Interestingly, 12 subsp. *septica* sequences contained the specific SNV C→T at position 520 in the *srlB* gene, that resulted in a premature stop codon. The one subsp. *septica* sequence that did not contain this SNV had a missense mutation, C→A at position 226, in the same gene. Therefore, it is likely that the *srl* operon is the gene associated with sorbitol fermentation in *P*. *multocida*, and specific SNVs are responsible for the lack of sorbitol fermentation in subsp. *septica* isolates. Further investigation is needed on a larger set of genomes of known subspecies. However, this serves as the first step towards development of an *in-silico* method for distinguishing *P*. *multocida* subspecies.

The *P*. *multocida* pangenome was also searched for genes associated with dulcitol (galactitol) fermentation, and the galactitol-specific PTS enzymes *gatA*, *gatB*, and *gatC*, were identified. Three of the four genomes of the *gallicida* subspecies contained *gatABC*, compared to just one of 63 *multocida* and *septica* genomes. However, this finding was not significant due to the small sample size of *gallicida* genomes compared to the other subspecies (OR = 63.0; *p* = 0.88). The association between *gatABC* and the *gallicida* subspecies warrants further analysis with a larger sample size and may eventually be used in combination with *srlAEBD* or genes not yet identified to distinguish all three *P*. *multocida* subspecies *in-silico*.

### Acquired antimicrobial resistance genes have integrated into *P*. *multocida* chromosome through mobile genetic elements

Acquired AMR-encoding genes conferring resistance to 11 different antimicrobial drug classes were identified among the *P*. *multocida* genomes (Table 3). The most common drug classes with resistance genes identified included aminoglycosides, with 120 (18.1%) *P*. *multocida* genomes containing an aminoglycoside resistance gene, followed by tetracyclines (17.5%), streptogramins (10.3%), macrolides (10.1%), and lincosamides (10.0%). Drug classes with fewer AMR genes identified included sulfonamides (9.2%), phenicols (7.6%), and beta-lactams (2.9%). Less than one percent of genomes contained genes conferring resistance to rifamycin (0.5%), fosfomycin (0.3%), and trimethoprim antibiotics (0.3%). Many of these genes were associated with mobile genetic elements determined using PlasmidFinder and ICEBerg 2.0 databases [56,57].

**Table 3:** Prevalence of antimicrobial resistance genes by drug class across 656 *Pasteurella multocida* genomes used in this study.

Class	No.	%	Genes
Aminoglycosides	120	18.1	*aadA1; aadA25; aadA31; aadK; ant(2’’)-Ia; aph(3’’)-Ib; aph(3’)-Ia; aph(6)-Id*
Beta-lactams	19	2.9	*bcII; bla1; blaA; bla*_*ORR-1*_*; bla*_*OXA-1*_*; bla*_*OXA-2*_*; blaP; bla*_*ROB-1*_*; bla*_*TEM-1*_*; bla*_*VEB-18*_
Fosfomycin	2	0.3	*fosA; fosB*
Lincosamides	66	10.0	*msrE; erm42*
Macrolides	67	10.1	*mefB; mphE; msrE; erm42*
Phenicols	50	7.6	*catA2; catA3; catD; cmlA5; floR; msrE*
Rifamycin	3	0.5	*arr-2; rphC*
Streptogramins	68	10.3	*erm42; satA; satB; msrE*
Sulfonamides	61	9.2	*sul2; sul3*
Tetracyclines	116	17.5	*msrE; tetA; tetB; tetH; tetQ; tetY*
Trimethoprim	2	0.3	*dfrA1; drfA32*

For example, the integrative and conjugative element (ICE), *ICEP*mu1, was identified in 73 isolates of bovine origin from the United States belonging to ST79 and one isolate of bovine origin with an unknown ST and geographic location. All genomes containing ICE*Pmu*1 aligned to the ICE*Pmu*1 reference sequence with >90% identity and >50% coverage. Interestingly, ICE*Pmu*1 was also identified in one *P*. *multocida* isolate from a turkey with an unknown sequence type. All genomes containing ICE*Pmu*1 belonged to serotype A. Notably, ICE*Pmu*1 contains the genes *aph(3”)-lb*, *aph(3’)-la*, *aph(6)-ld*, conferring resistance to aminoglycoside drugs, *erm(42)*, conferring resistance to macrolide drugs, *tetH*, conferring resistance to tetracycline, and *sul2*, conferring resistance to sulfonamides.

**Fig 3:**
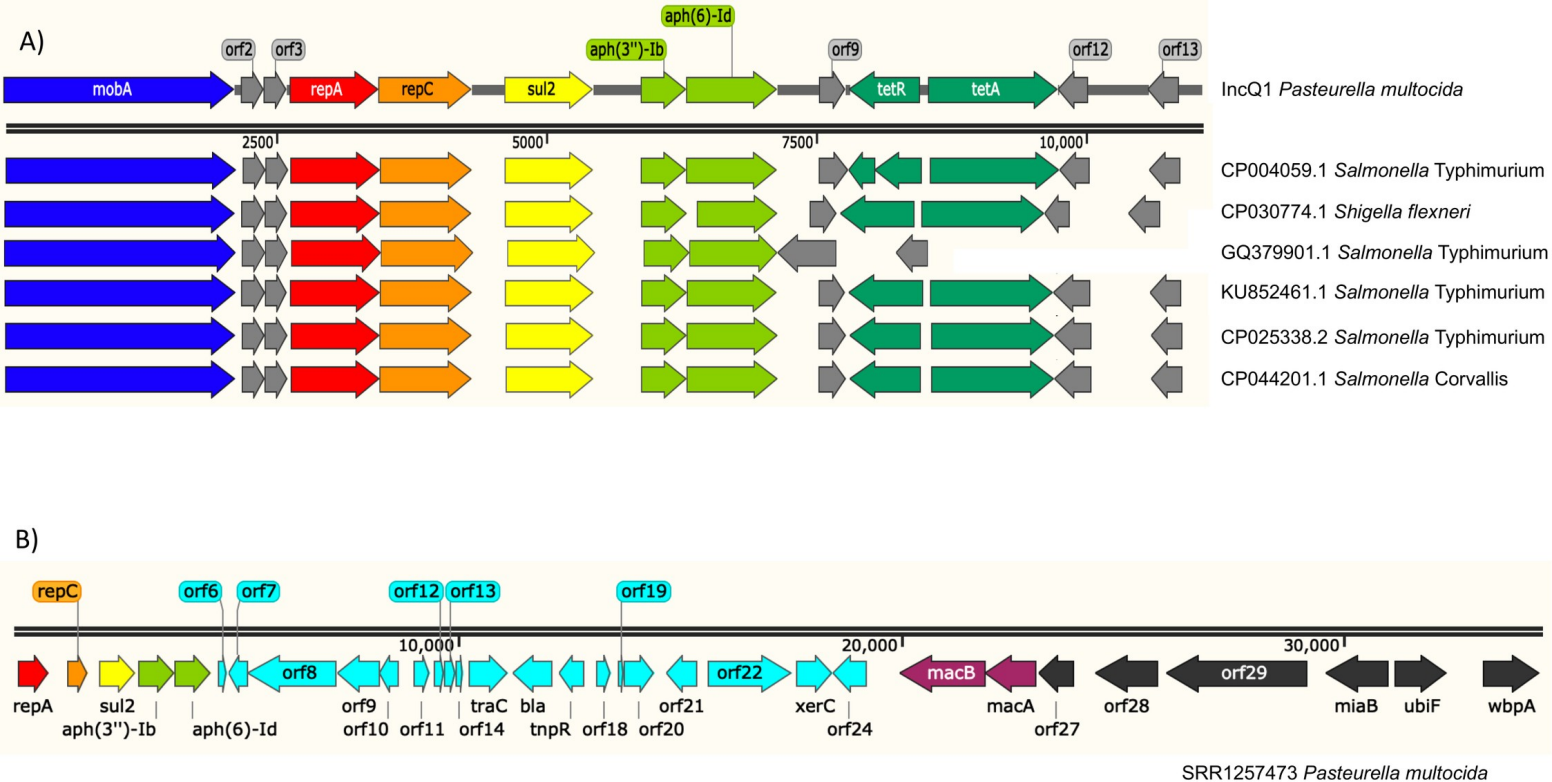
IncQ1 plasmid maps and chromosomal insertion. A) Map of IncQ1 plasmids from *P*. *multocida* compared to IncQ1 plasmids of other pathogenic bacterial species. B) Partial insertion of IncQ1 plasmid into the *P*. *multocida* chromosome in a isolate of bovine origin, adjacent to ICE*hin1056*. Dark blue = mobilization gene; red and orange = replication genes; yellow = sulfonamide resistance gene; light green = aminoglycoside resistance genes; dark green = tetracycline resistance genes; light blue = ICE*hin1056;* mauve = macrolide resistance genes.

IncQ1 plasmids were identified in six *P*. *multocida* isolates of avian origin from North America. This plasmid also contains *aph(3”)-lb*, *aph(6)-ld*, and *sul2*, and additionally includes two tetracycline genes, *tetA* and *tetR*. These IncQ1 plasmids shared >99% nucleotide identity with IncQ1 plasmids identified in other pathogenic bacterial species, including *Salmonella enterica* subsp. Typhimurium and *Shigella flexneri* (Fig 3A). IncQ1 replicons were also identified in six isolates of bovine origin from Asia. However, upon further inspection, only a fragment of the entire plasmid was identified in each of these genomes, and it appeared to be inserted into the chromosome at similar locations in all cases. Adjacent to the insertion was an ICE which had >99% nucleotide identity with ICE*hin1056* of *Haemophilus influenzae*. Annotation of ICE*hin1056* revealed the presence of additional antimicrobial resistance genes including *bla*_TEM-1_, which confers resistance to beta-lactam antibiotics (Fig 3B). All genomes that included a partial or whole IncQ1 plasmid were the same sequence type, ST122, which suggests that IncQ1 was potentially acquired in a single event, and possibly a second event integrated this element into the chromosome.

### Distribution of virulence factors demonstrates association with specific capsular serogroups

Genomes were searched for the presence of 15 known *P*. *multocida* virulence factors (Table 4). All 656 genomes contained Omp16, TbpA, and PlpB. More than 99% of genomes also contained HgbB, OmpH2, ComE, PlpD, and PfhB2, while less than 99% of genomes contained HgbA, OmpH1, OmpH3, PtfA, PlpE, PlpP, and PfhB1. These genes were further investigated for statistically significant associations with capsular serogroups A, B, D, and F (Table 5). Capsular serogroup B genomes had significantly increased odds (OR = 29.07; *p*<0.00001) of containing HgbA, Hemoglobin-binding protein A, compared to genomes of all other capsular serogroups. Capsular serogroup A genomes had significantly increased odds of containing the outer membrane proteins OmpH1 (OR = 9.09; *p*<0.00001) and OmpH3 (OR = 2.56; *p* = 0.00003) compared to all other capsular serogroups. PtfA, a Type IV Fimbriae, was significantly associated with capsular serogroups B (OR = infinite; *p* = 0.00272) and F (OR = 7.9; *p* = 0.00734). PlpE and PlpP, protective outer membrane lipoproteins, were significantly associated with capsular serogroups A (OR = 4.25; *p*<0.00001) and F (OR = 8.96; *p*<0.0001), respectively. Lastly, capsular serogroup A genomes had statistically increased odds of containing, PfhB1, a filamentous hemagglutinin protein, compared to all other genomes (OR = 3.64; *p*<0.00001).

**Table 4:** Prevalence of select virulence factors across 656 *Pasteurella multocida* genomes in this study.

Protein	Acc. No.	Description	No.	%
HgbA	AAQ14873.1	Hemoglobin-binding protein A	240	36.59
HgbB	APX53047.1	Hemoglobin-binding protein B	650	99.09
Omp16	AHW46109.1	Outer membrane protein 16	656	100.00
OmpH1	SNV59447.1	Outer membrane protein H1	113	17.23
OmpH2	VEE38334.1	Outer membrane protein H2	652	99.39
OmpH3	AMK08231.1	Outer membrane protein H3	386	58.84
TbpA	AAK02460.1	Transferrin binding protein A	656	100.00
PtfA	AKO69808.1	Type IV fimbriae	530	80.79
ComE	AFF25459.1	Response regulator	650	99.09
PlpE	AAK03601.1	Protective outer membrane lipoprotein E	453	69.05
PlpP	AAK03602.1	Protective outer membrane lipoprotein P	180	27.44
PlpB	AAK03814.1	Protective outer membrane lipoprotein B	656	100.00
PlpD	VEE38139.1	Protective outer membrane lipoprotein D	651	99.24
PfhB1	AAK02141.1	Filamentous hemagglutinin protein B1	482	73.48
PfhB2	AAK02143.1	Filamentous hemagglutinin protein B2	650	99.09

**Table 5:** Association between *Pasteurella multocida* virulence factors and capsular serogroups in this study (OR = odds ratio, CI = confidence interval, Inf = infinity).

	OR	95% CI	*p*-value
**HgbA**			
A	0.68	(0.46, 1.03)	0.060
B	29.07	(9.11, 148.51)	<0.001
D	0.37	(0.12, 0.93)	0.720
F	0.43	(0.21, 0.83)	0.226
**OmpH1**			
A	9.09	(3.35, 34.59)	<0.001
B	0.00	(0.00, 0.38)	0.008
D	0.14	(0.00, 0.87)	0.832
F	0.22	(0.04, 0.71)	0.119
**OmpH3**			
A	2.56	(1.72, 3.83)	<0.001
B	0.00	(0.00, 0.06)	<0.001
D	0.73	(0.34, 1.58)	1.00
F	1.30	(0.73, 2.36)	1.00
**PtfA**			
A	0.09	(0.03, 0.26)	<0.001
B	Inf	(2.98, inf)	0.003
D	3.85	(0.96, 33.61)	1.00
F	7.90	(2.04, 67.76)	0.007
**PlpE**			
A	4.25	(2.83, 6.42)	<0.001
B	0.02	(0.00, 0.06)	<0.001
D	0.15	(0.06, 0.35)	0.001
F	1.77	(0.92, 3.65)	1.00
**PlpP**			
A	0.75	(0.49, 1.15)	1.00
B	0.00	(0.00, 0.21)	0.00001
D	0.00	(0.00, 0.29)	0.001
F	8.96	(4.84, 17.28)	<0.001
**PfhB1**			
A	3.64	(2.41, 5.52)	<0.001
B	0.01	(0.00, 0.05)	<0.001
D	2.71	(0.93, 10.80)	1.00
F	0.68	(0.38, 1.25)	1.00

## Discussion

This large-scale bioinformatics study provides a comprehensive overview of the molecular epidemiology of *P*. *multocida* using publicly available whole-genome sequence data. Despite the broad host range of isolates in this study, there is little evidence for host predilection within *P*. *multocida*. This is apparent in MLST results, as well as the core SNV-based phylogenetic tree, as isolates from different hosts were found to belong to the same sequence type, and host types were distributed throughout the phylogenetic tree. This finding is similar to the results of other genotypic studies of *P*. *multocida* that compared different host types, but only assessed the seven MLST genes [24,37,45]. There does seem to be an exception, however, with isolates from bovine hosts. Previous studies have demonstrated a clonal population structure of *P*. *multocida* isolates associated with hemorrhagic septicemia in cattle [38,58,59]. Although there was limited information available on disease status for the isolates in this study, it is evident that almost all of the 155 isolates from bovine sources fell into specific phylogenetic clades that were clustered together on the tree. Interestingly, this specificity is demonstrated further when looking at the individual bovine hosts, as 28 of the 30 isolates from buffalo belong to ST122, with collection dates ranging from 2003 to 2016. It should be acknowledged, however, that there are two different MLST schemes for *P*. *multocida*, limiting direct comparisons between studies that used different schemes. Additionally, the lack of host-specificity identified in this study may be limited by the data available in the NCBI SRA and Assembly databases. For example, in this study, ST20 genomes were exclusively from chicken isolates, but this sequence type has also been identified in a *P*. *multocida* isolate from a pig in Australia [24].

A lack of host-specificity was also noted within *P*. *multocida* capsular serogroups, as all but three host categories listed in Table 2 had genomes fall into multiple capsular serogroups. Four different capsular serogroups were identified in isolates of avian and swine origin, and three different capsular serogroups were identified in isolates of bovine and rabbit origin. This is contradictory with some reports in the literature that suggest *P*. *multocida* serogroups display host predilection [2,60–62]. There was also no evidence of a geographic distribution of specific capsular serogroups. Previous studies have claimed that certain geographic regions have increased prevalence certain serogroups, however, these studies were limited in scope and sample size [2,63,64]. The results of this study indicate that, outside of bovine sources, there is an overall lack of host specificity in relation to capsular serogroup and sequence type, as well as a lack of geographical associations with certain capsular serogroups.

Genomes of different capsular serogroups were dispersed throughout the phylogenetic tree. While genomes within particular clades mostly belonged to the same capsular serogroup, the overall distribution of those genomes on the tree indicates that differences in capsular serogroups may not always correspond to differences at the whole-genome level. One possible explanation could be homologous recombination of the capsule genes. During the SNV analysis, there were 82 recombination events detected in the region of the genome containing the capsule genes that were removed from the SNV alignment. Therefore, SNVs identified in the capsule genes that determine serogroup were not incorporated into the resulting phylogenetic tree. While this study did not include somatic serotyping, it has been shown that somatic serotyping is also a poor indicator of the genetic relatedness of *P*. *multocida* isolates [65].

Specific virulence genes demonstrated significant associations with particular capsular serogroups. For example, while HgbA was found in genomes of all serotypes, the odds of HgbA being present in capsular serogroup B genomes was 29 times higher compared to all other capsular serogroups. This contrasts with previous studies that found HgbA is almost always present among *P*. *multocida* isolates regardless of capsular serogroup or host [66–70]. PtfA, a Type IV fimbriae, was also associated with capsular serogroup B, and has previously shown to be an important factor in disease outcomes in cattle [67]. This protein was also significantly associated with capsular serogroup F. OmpH1, an outer membrane protein, was not identified in any genomes from capsular serogroup B, and was significantly associated with capsular serogroup A. However, a previous study has indicated that the *ompH1* genes of *P*. *multocida* have undergone extensive horizontal gene transfer, as well as intragenic and assortative recombination [71]. In this study, it appears that different alleles of the gene encoding OmpH1 exist in other capsular serogroups with enough sequence variation that they did not meet the criteria for containing OmpH1, as many genomes had blast identity scores in the 70–90% range. OmpH3 was not identified in any capsular serogroup B genomes and was significantly associated with capsular serogroup A. The protective outer membrane lipoprotein PlpE was also significantly associated with capsular serogroup A. However, PlpP was significantly associated with capsular serogroup F. In fact, capsular serogroup F was the only capsular serogroup with an odds ratio greater than one. These findings warrant further investigation into the diversity and prevalence of OmpH and Plp proteins in *P*. *multocida*, as these surface antigens are often used in vaccine studies that attempt to elicit protective immunity against *P*. *multocida* infection [72–76].

The SNV analysis on a subset of isolates involved in fowl cholera outbreaks from a single turkey producer revealed that multiple *P*. *multocida* strains may be identified in a single outbreak, which also has implications for vaccination. There are live-attenuated vaccines available for the prevention of fowl cholera in turkeys, as well as adjuvant or autogenous bacterins. The choice of vaccine type is typically dependent on bird age, stage of production, and which strains are currently circulating in the flocks of interest. Traditional *P*. *multocida* serotype designations are very broad, whether it be the Carter capsular serogroups, Heddleston somatic serovars, or the combination of both, so serological data alone is often not enough to distinguish specific strains to use for vaccination. This emphasizes the importance of more discriminatory methods, such as whole-genome sequencing, when making decisions about vaccine strategy. However, whole-genome sequencing may not be readily available or economically feasible in many cases. Alternative typing approaches have been developed in recent years, including PFGE and REP-PCR, which may provide a more granular approach to classifying *P*. *multocida* isolates [77–79].

This study is the first to provide genotypic evidence for differentiation between *P*. *multocida* subspecies with the identification of specific mutations in the *srlB* gene in subsp. *septica* genomes. While these mutations could distinguish subsp. *septica* from *multocida* and *gallicida*, subsp. *multocida* and *gallicida* could not be distinguished from one another through the genomic approaches utilized in this study. A previous study suggested the possibility of two distinct lineages of *P*. *multocida* based on subspecies: subsp. *multocida* and *gallicida* as one, and subsp. *septica* as the other [39]. However, in the present study, different subspecies, including *septica*, were identified as the same sequence type, such as with ST9 and ST129, which included both *multocida* and *septica* genomes. Further, genomes of the same subspecies were found throughout the phylogenetic tree instead of in clusters as one would expect of distinct lineages. This needs further investigation, however, as this study could readily distinguish *septica* genomes with SNVs in the *srlB* gene but were unable to distinguish subsp. *multocida* and *gallicida* using similar methodology. These findings also call into question the overall value of the information provided by subspecies distinction, as different hosts, capsular serogroups, and sequence types have been found within the same subspecies, and subspecies did not correspond to whole-genome differences in this study. Although subspecies differentiation may have provided early insight into the microbiology of *P*. *multocida* before other typing methods were developed, the identification of two sugar utilization systems has limited application in the context of genomic or epidemiological studies.

Several mobile genetic elements were identified within the *P*. *multocida* genomes in this study, some of which were highly similar to those from other bacterial species. This was particularly evident with the IncQ1 plasmids identified, as almost identical plasmids were found in strains of *Salmonella* Typhimurium, *Salmonella* Corvallis and *Shigella flexneri*. The match with the highest percent identity to *P*. *multocida* IncQ1 plasmids was a plasmid of *Salmonella* Typhimurium U288 (CP004059.1), a significant pathogen of pigs in the United Kingdom [80]. Other similar IncQ1 plasmids were from a human *Salmonella* Typhimurium (KU852461.1) clinical isolate from Southern Italy, a human *Shigella flexneri* (CP030774.1) clinical isolate from Bangladesh, and a *Salmonella* Corvallis isolate (CP044201.1) from an unknown source in the United States [81]. In this study, the *P*. *multocida* genomes containing the entire IncQ1 plasmid were from poultry isolates in the United States, demonstrating that the dissemination of IncQ1 plasmids has not been hindered by host or geography. This is notably concerning due to the presence of antimicrobial resistance genes, as well as the potential to integrate into the bacterial chromosome. Additionally, the identification of this plasmid in isolates from poultry is also concerning given that this IncQ1 plasmid contains *sul2*, *tetA*, and *tetR*, conferring resistance to sulfonamide and tetracycline drugs, both of which have been used for prevention and treatment of fowl cholera in poultry in the United States [82].

The identification of antimicrobial resistance genes, virulence genes, and mobile genetic elements in this study provide important epidemiological context to the various typing methods often used in *P*. *multocida* diagnostics and research. The use of whole-genome sequence data has enhanced the overall understanding of the relationships, or lack of relationships, between host, geography, specific genotypes, and phylogeny in *P*. *multocida*. This study relied on sequences and metadata available in NCBI databases, which allowed for the large sample size necessary to make appropriate statistical associations in most cases, but was not without limitations. Information on host, geographic location, and collection date were not available for all genomes, and only a limited number of genomes had information on capsular serogroup or subspecies. Additionally, there were several *P*. *multocida* genomes with low coverage of the *cap* genes used to distinguish capsular serogroups. A larger set of *P*. *multocida* genomes of known capsular serogroups are needed to explore this further and validate the *in-silico* methodology developed in this study. It was assumed that all of the information that accompanied the genome sequences in the NCBI databases was correct. The overrepresentation of isolates of avian origin, as well as capsular serogroup A genomes, and isolates from the United States, should be acknowledged. The addition of 125 genomes from the University of Minnesota Midwest Central Research and Outreach Center contributed to this overrepresentation, as did batch uploads within the NCBI databases that allowed for the submission of multiple sequences at the same time by a single user. Therefore, the genomic diversity demonstrated in this study may actually underrepresent the true genomic diversity of *P*. *multocida*. However, this is the nature of large-scale bioinformatic studies and does not undermine the considerable insight gained from using publicly available data. This study expanded the current understanding of *P*. *multocida* molecular epidemiology through comparative genomics of 656 whole-genome sequences that varied in host and geographic origins. The genomic landscape of *P*. *multocida* demonstrates ample genetic diversity and has elucidated patterns in the potential for virulence and antimicrobial resistance that can be used to inform researchers and clinicians about factors that may influence the course of *P*. *multocida* infections.

## Methods

### Genome download, assembly, and multi-locus sequence typing

All available *P*. *multocida* whole-genome sequences and associated metadata were downloaded from the NCBI Short Read Archive and NCBI Assembly databases using the SRA toolkit v. 2.9.1 and NCBI-genome-download v. 0.2.7 [83,84]. These genomes were combined with a set of 125 *P*. *multocida* whole-genome sequences from the University of Minnesota Mid-Central Research and Outreach Center (Willmar, MN, USA) (BioProject PRJNA666906). The *P*. *multocida* genomes generated during this study came from clinical isolates of fowl cholera from turkeys within a single turkey production company. DNA was extracted using the Qiagen DNeasy blood and tissue kit (Valencia, CA). The genomic library was produced using the Nextera XT library prep kit and Nextera XT index kit v. 2 (Illumina, San Diego, CA) according to the manufacturer’s instructions. Sequencing was performed on an Illumina MiSeq system using a 2 x 250 dual-index approach. Raw Illumina reads were trimmed of adaptor content and filtered for quality using Trimmomatic v. 0.33 using default settings [85]. For genomes that were already assembled from the NCBI Assembly database, raw Illumina reads were simulated using Wgsim v. 1.0.2 and then trimmed using the same methods (https://github.com/lh3/wgsim). All genomes were assembled with Spades v. 3.10.0 using default settings [86]. Genome assemblies were assessed for quality using QUAST v. 4.3 with reference genome ATCC 43137 (GCA_000754275.1) [87]. Genomes were excluded from subsequent analysis if the genome fraction (i.e. proportion of aligned bases from the reference) was less than 80%, which would indicate that the genome may be of a species other than *P*. *multocida*. Genomes were also excluded if the number of unaligned bases in the assembly was greater than 2 million, which would be indicative of possible contamination by a species other than *P*. *multocida*. Multi-locus sequence typing was performed on the remaining *P*. *multocida* genomes using the RIRDC scheme available in the PubMLST database (https://github.com/tseemann/mlst) [32,33,88]. This scheme was selected because it contains a larger number of sequence types that would ideally reduce the number of non-typable genomes in this study.

### *In-silico* identification of *P*. *multocida* capsular serogroups

There are genes unique to specific capsular serogroups (*hyaD*, *bcbD*, *dcbF*, *ecbJ*, and *fcbD*) within the capsular biosynthetic loci of *P*. *multocida*. These genes have previously been used in a multiplex PCR assay that was successful in distinguishing *P*. *multocida* capsular serogroups [89]. The nucleotide sequences for these genes unique to capsular serogroups A (AF067175), B (AF169324), D (AF302465), E (AF302466), and F (AF302467) were downloaded from GenBank and used to construct a custom blast database using BLAST+ v. 2.8.1 [90,91]. This database was used in a nucleotide blast of all genome assemblies in order to identify top hits to the genes encoding the capsular antigens. Hits for each genome with a bitscore of less than 1,000 were removed. Of the remaining top hits, the one with the highest percent identity was selected as the capsular serogroup for that genome. This methodology was validated by comparing the *in-silico* capsular serogroup results to the capsular serogroup information available in the metadata downloaded from NCBI. All custom databases generated in this study are available on GitHub (https://github.com/JohnsonSingerLab/Pmult-databases).

### Single nucleotide variant (SNV) analysis

Snippy v. 4.6.0 was used to call core SNVs from the trimmed reads of each genome and generate a core SNV alignment using default parameters with reference genome ATCC 43137 (GCA_000754275.1) [92]. Gubbins v. 2.3.4 was used to remove SNVs found in recombinant regions from the alignment [93]. The ModelFinder tool within IQ-TREE was used to identify the optimal model of DNA substitution for estimating maximum-likelihood phylogeny [94,95]. A phylogenetic tree was constructed using the selected model of DNA substitution with a correction for constant sites determined using maskrc-svg (https://github.com/kwongj/maskrc-svg) and snp-sites [96]. An Ultrafast bootstrap approximation with 1,000 replications was performed [97]. The resulting tree was annotated using iTOL [98].

### *P*. *multocida* pangenome and differences in gene content

Genomes were annotated using Prokka v. 1.13, and Roary v. 3.10.2 was used to construct the *P*. *multocida* pangenome using default parameters [99,100]. Scoary v. 1.6.16 was used to identify significant differences in gene content between phylogenetic clades with Fisher’s exact tests and the Benjamini-Hochburg correction to control the false discovery rate [101,102]. Odds ratios and *p*-values are reported, with a *p*-value of ≤0.05 considered significant.

The pangenome was also used to search for genes that may be associated with the ability to ferment sorbitol and dulcitol in order to identify any associations with *P*. *multocida* subspecies. Subspecies information was only available for a limited number of the *P*. *multocida* genomes in this study (*n* = 68). SNVs were identified using the same methods described previously, with the reference genome consisting of the gene sequences extracted from the pangenome reference file. SNVs within each alignment were assessed for missense or nonsense mutations that could potentially alter the function of the gene.

### Identification of antimicrobial resistance genes, mobile genetic elements, and virulence factors

Antimicrobial resistance genes were identified from the genome assemblies using Abricate v. 0.9.8 with the NCBI AMRFinder database [103,104]. Genes with coverage of less than 50% were excluded. Plasmid replicons were identified from the genome assemblies using Abricate v. 0.9.8 with the PlasmidFinder database [56,103]. When specific replicons were identified, a nucleotide blast search was performed to determine if the whole plasmid was present. Plasmid sequences were circularized, oriented to the same start site, aligned with Mauve, and visualized using SnapGene Viewer (from GSL biotech; available at snapgene.com) [105].

To identify genomes with the known integrative conjugative element associated with *P*. *multocida*, ICE*Pmu*1, a blast search was conducted on genome assemblies using the ICE*Pmu*1 reference sequence from ICEberg 2.0 [57,91,106]. Genomes that aligned to ICE*Pmu*1 with at least 50% coverage and 90% identity were considered to contain ICE*Pmu*1. This included genomes with alignments on multiple contigs, provided that 50% of the reference sequence was still covered.

A blast search was conducted for a set of virulence factors that differ in prevalence among virulent strains of *P*. *multocida* as determined in a prior study [68,91]. The annotated amino acid sequences of each genome were searched for these virulence-associated proteins. Sequences producing alignments with greater than 50% coverage and 90% identity were considered to contain the virulence factor. To test whether these virulence factors were associated with specific capsular serogroups, Fisher’s exact tests with the Benjamini-Hochburg correction were performed in R v. 1.2.5042 [102,107]. Odds ratios, confidence intervals, and *p*-values are reported.

## Supporting information

S1 Dataset (XLSX)
